# Addressing Functional Mitral Regurgitation in Dilated Cardiomyopathy: A Focus on Myocardial Segment Resynchronization Through Cardiac Resynchronization Therapy

**DOI:** 10.7759/cureus.74374

**Published:** 2024-11-24

**Authors:** Aysegul Karahan, Enis Oguz

**Affiliations:** 1 Department of Cardiology, Liv Hospital Ulus, Istanbul, TUR

**Keywords:** cardiac resynchronization therapy, dilated cardiomyopathy, functional mitral regurgitation, left bundle branch block, papillary muscle dyssynchrony

## Abstract

Introduction: Cardiac resynchronization therapy (CRT) has emerged as a pivotal intervention in reducing functional mitral regurgitation (FMR), not only by enhancing global left ventricular (LV) systolic function but also by refining local myocardial synchronization. This study hypothesized that CRT-mediated synchronization of myocardial segments, particularly between papillary muscles, reduces FMR further, independent of the improvement of the LV systolic indices.

Methods: Eighteen patients with dilated cardiomyopathy and biventricular pacing were evaluated. Measurements included the rate of rise in LV systolic pressure (LV dP/dt), asynchrony indices, transmitral pressure differences, mitral regurgitation quantification and diastolic filling times during pacing and with pacemaker interruption. As LV dP/dt decreased while the pacemaker was interrupted, dobutamine infusion was administered to restore LV dP/dt to pacing levels. All parameters were reassessed to evaluate the impact of myocardial resynchronization on FMR, independent of LV systolic performance.

Results: LV dP/dt significantly decreased in 10 patients after pacemaker interruption (838±190 vs 444±72, p<0.01), with no significant change in eight patients (603±134 vs 592±156, p=0.679). Despite similar LV performance indices, biventricular pacing led to a statistically significant reduction in both effective regurgitant orifice area and regurgitant volume across all patients (p<0.001 and p=0.003, respectively).

Conclusion: CRT significantly reduces FMR in dilated cardiomyopathy patients with intraventricular delay independent of improvements in LV systolic performance indicators by resynchronization of the LV segments underlying the papillary muscles. Moreover, it may be the main determinant of the reduction in FMR in CRT, underscoring the need for further research into its mechanisms and therapeutic implications.

## Introduction

Functional mitral regurgitation (FMR) is a common complication of left ventricular (LV) failure and remodeling in patients with ischemic or nonischemic dilated cardiomyopathy (DCM) [1]. It develops because of an imbalance between the tethering and closing forces of the mitral valve leaflets. Systolic valvular tenting, which results from apical and posterior papillary muscle displacements, is well known to be the primary determinant of FMR along with diminished LV contraction and mitral annular dilatation playing significant roles [2-4]. On the other hand, a co-determinant of FMR is intra- and atrioventricular dyssynchrony. Epidemiologic registries have revealed that QRS widening is relatively frequent (21-47%) in patients with DCM with the conduction delay often presenting as left bundle branch block (LBBB) and FMR severity correlates with QRS duration in such patients [5,6]. LBBB, by decreasing LV closing forces, increasing tethering forces, impairing mitral annulus function, and increasing atrioventricular dyssynchrony, increases the severity of FMR [7].

Enhancement of LV function through cardiac resynchronization therapy (CRT) in patients with DCM leads to an increased rate of rise in LV systolic pressure (LV dP/dt), thereby augmenting the transmitral pressure gradient and mitigating the tenting forces that hinder mitral valve closure, ultimately resulting in a reduction of FMR [8]. On the other hand, dyssynchronization of the medial and lateral segments that support the papillary muscles may increase the degree of FMR in patients with heart failure, independent of the other factors. Many studies have shown that the severity of FMR was correlated with the papillary muscle dyssynchrony [9-12], and CRT reduces FMR not only by increasing closing forces but more importantly through local synchronization [13-15].

In this study, we hypothesize that the observed reduction in FMR with CRT is predominantly attributable to the correction of dyssynchrony between the papillary muscles, rather than being solely due to enhancements in global LV systolic performance. To investigate this, we administered dobutamine perfusion to patients whose LV dP/dt significantly decreased upon interruption of biventricular pacing, aiming to restore dP/dt to levels achieved during pacing as dobutamine is known to enhance dP/dt and closing forces [16]. Subsequently, FMR degree and transmitral pressure differences of the patients during the isosystolic pressure rise rate were re-evaluated. This methodological approach allowed the study to isolate the effects of synchronization from general LV systolic improvements, thus providing a more precise understanding of CRT's mechanistic impact on FMR.

## Materials and methods

Patients

Thirty-two patients with DCM were included in our study. All our patients had severe LV systolic heart failure (transthoracic biplane ejection fraction average 22 ±5.6%), FMR detected by color flow Doppler and significant functional limitation (New York Heart Association (NYHA) class II/III). Patients were treated with diuretics, renin-angiotensin system (RAS) renin angiotensin receptor antagonists, mineralocorticoid receptor antagonists and beta-blockers as tolerated. All patients had biventricular pacemaker electrodes placed in the right ventricular apex and LV via the coronary sinus for CRT. As the primary objective of our study was to focus on the specific mechanistic contribution of local myocardial synchronization, particularly in segments supporting the papillary muscles we deliberately included patients who exhibited good response to CRT and showed a significant reduction in FMR. Therefore, non-responder patients were not included in the study. Additionally in 14 of 32 patients, LV dP/dt significantly decreased during the biventricular interruption and could not however be able to reach to pacing level with dobutamine infusion, therefore these patients were excluded from the study. The Ethics Committee of Siyami Ersek Thoracic and Cardiovascular Center issued approval 2005 and the study was conducted in accordance with the Declaration of Helsinki. Informed consent was obtained from all participants included in the study.

Echocardiographic protocol

**Figure 1:**
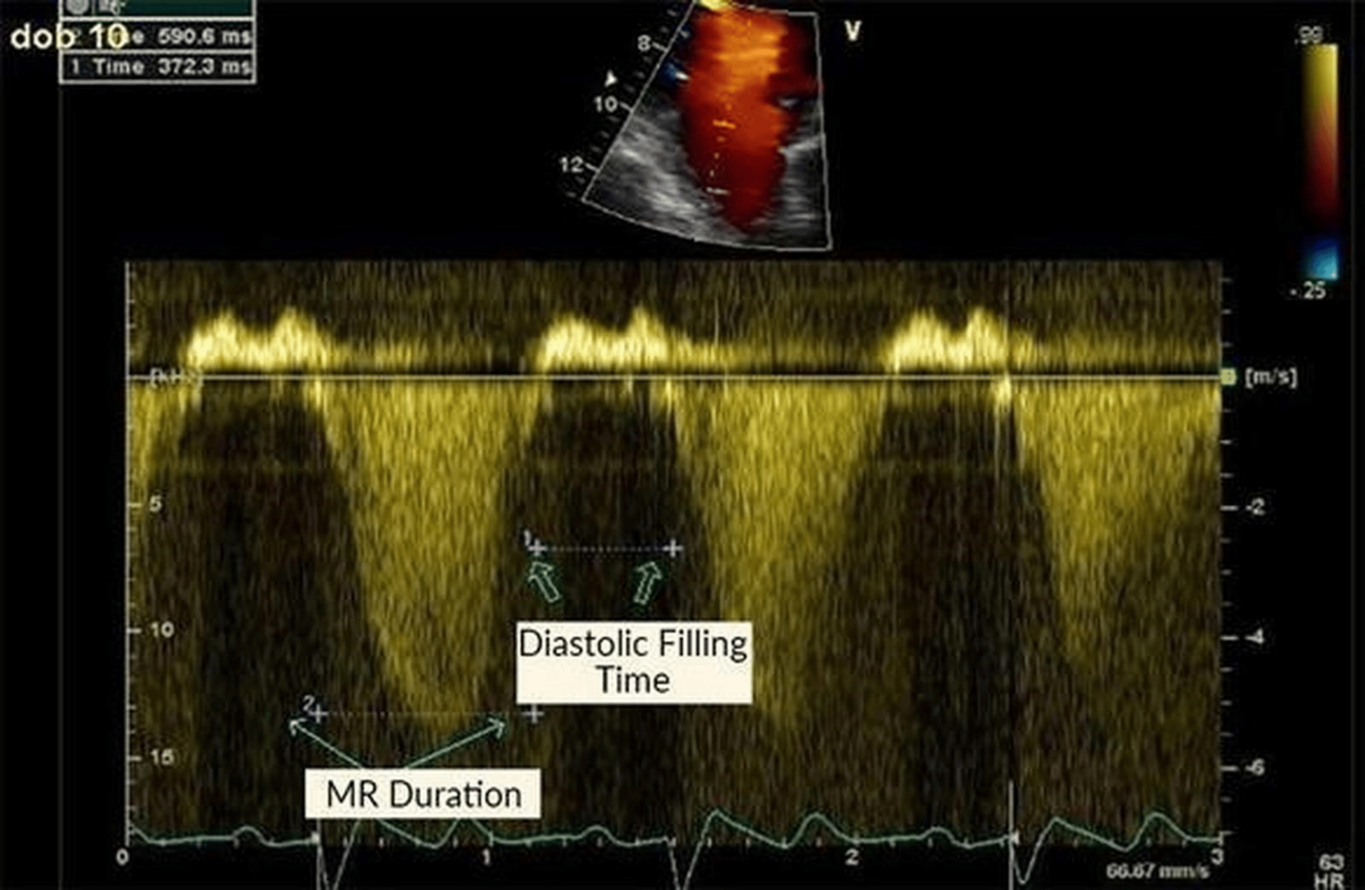
Measurement of mitral regurgitation (MR) duration and diastolic filling time.

Echocardiographic examinations were performed with a Vivid 7 Digital ultrasound device (Vingmed Ultrasound, GE, Horten, Norway) using a 2.5-3.5 MHz wide band transducer. LV diameters were measured in M-mode, guided by 2-D imaging. LV end-systolic and diastolic volumes and ejection fractions were calculated using Simpson's method from apical 4- and 2-chamber views. All patients underwent standard 2-D and Doppler echocardiographic examination in the left lateral supine position during biventricular pacing and pacemaker interruption. FMR was quantified by an integrated approach comprising mitral valve morphology, and the proximal isovelocity surface area (PISA) method, which includes the effective regurgitant orifice (ERO) and the regurgitant volume (RV) [17]. During biventricular pacing, EROs and RVs using color Doppler flow and PISA method, asynchrony indices with strain rate imaging, transmitral pressure differences with Bernoulli equation and mitral regurgitation and diastolic filling times with Doppler method were calculated in all patients (Figure 1).

As LV dP/dt decreased while the pacemaker was interrupted, dobutamine infusions were administered at 5, 10, 20, and 40 mcg/kg/min, respectively, to increase LV dP/dt to the pacing level. Dobutamine infusion was discontinued when the intended dP/dt was reached and all these parameters were reassessed. Programming of the biventricular pacemakers to on and off mode was done while the patient was stationary during the echocardiographic examination.

Left ventricular dP/dt measurement

**Figure 2:**
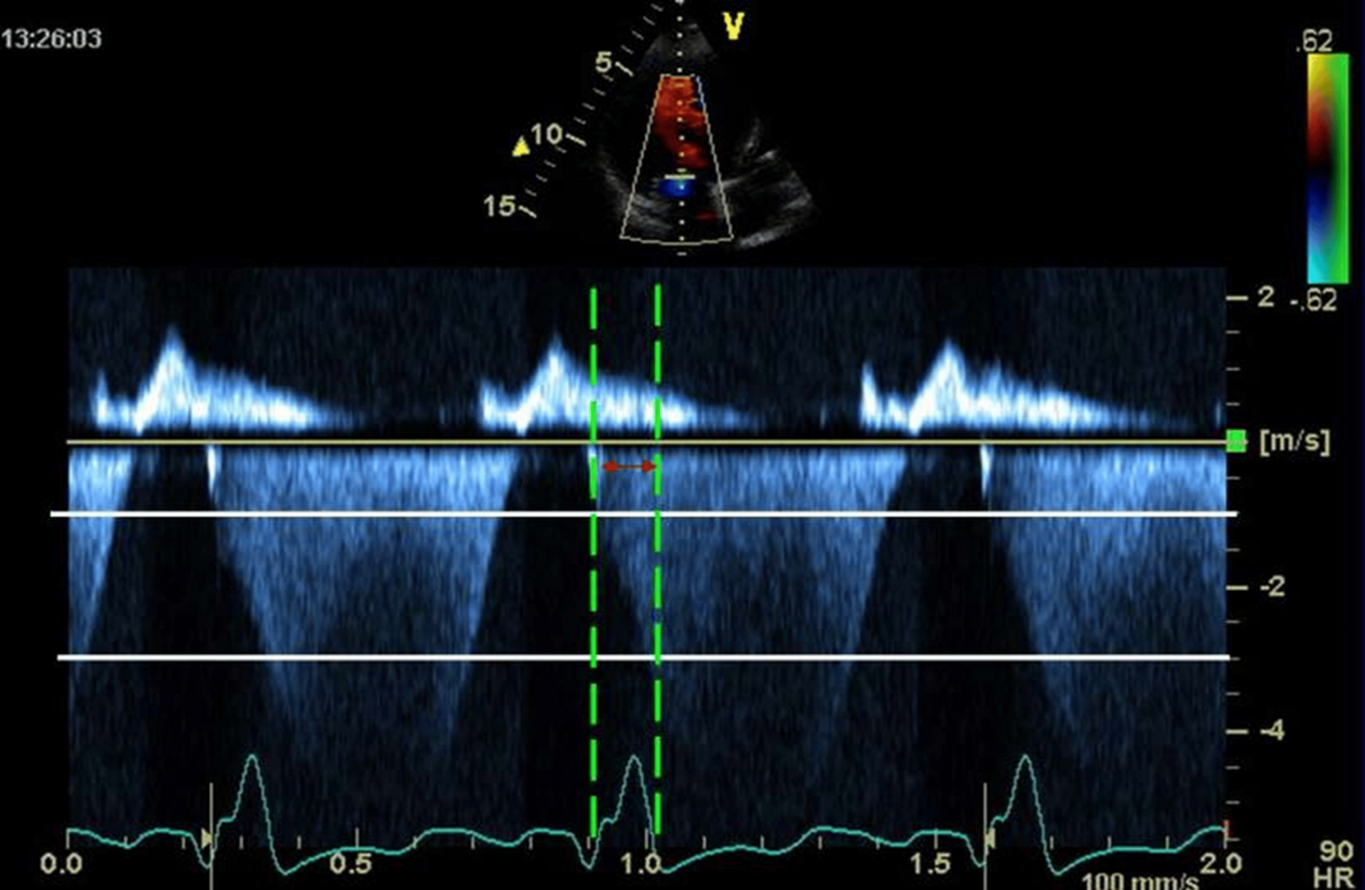
Measurement of left ventricular systolic pressure (LV dP/dt) from mitral regurgitation

Continuous-wave (CW) Doppler tracing of mitral regurgitation flow was obtained from apical 4- and 2-chamber views. The ultrasonic flow was carefully positioned to be parallel to the direction of the mitral regurgitation flow, and the "gain", compress, wall filter and velocity scale settings were optimally adjusted to obtain a smooth-edged spectral Doppler tracing. Mitral regurgitation time was measured and LV dP/dt was measured using the rate pressure rise method from the CW Doppler trace. Briefly, the time from 1 m/sec to 3 m/sec of velocity was measured in CW Doppler tracing. Then, the pressure difference between these velocities (P= 4V2) calculated according to Bernoulli's equation (36 mmHg-4mmHg) was divided into the measured time interval (Figure 2).

Transmitral pressure gradient measurement

**Figure 3:**
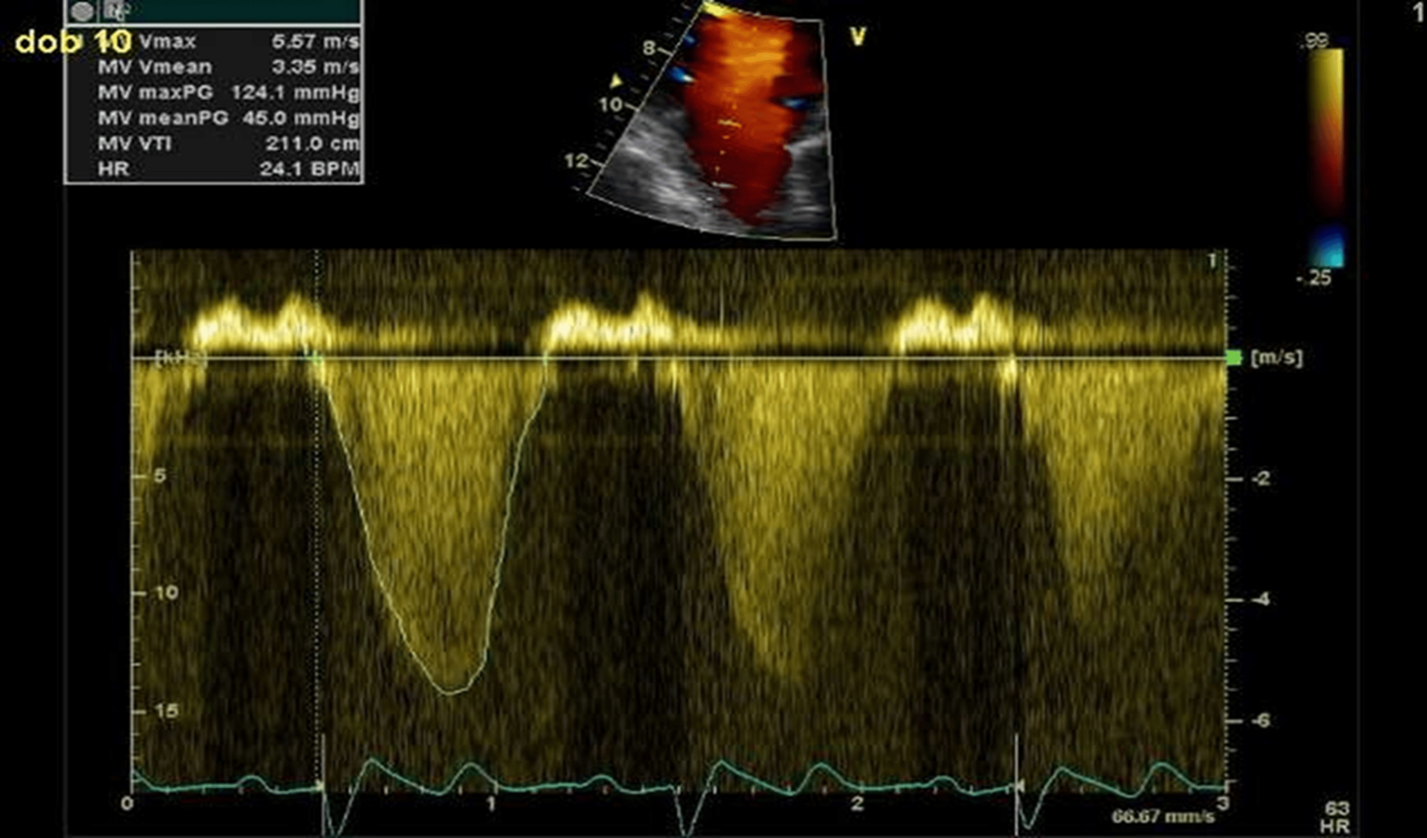
Measurement of transmitral pressure gradient

The transmitral pressure difference was defined as the difference between LV and left atrial pressures during systole. In the presence of mitral regurgitation, the maximum transmitral pressure difference is determined by the Bernoulli equation (4xMR Max2) from the CW Doppler trace of the mitral regurgitation jet (Figure 3).

Measurement of the asynchrony index

**Figure 4:**
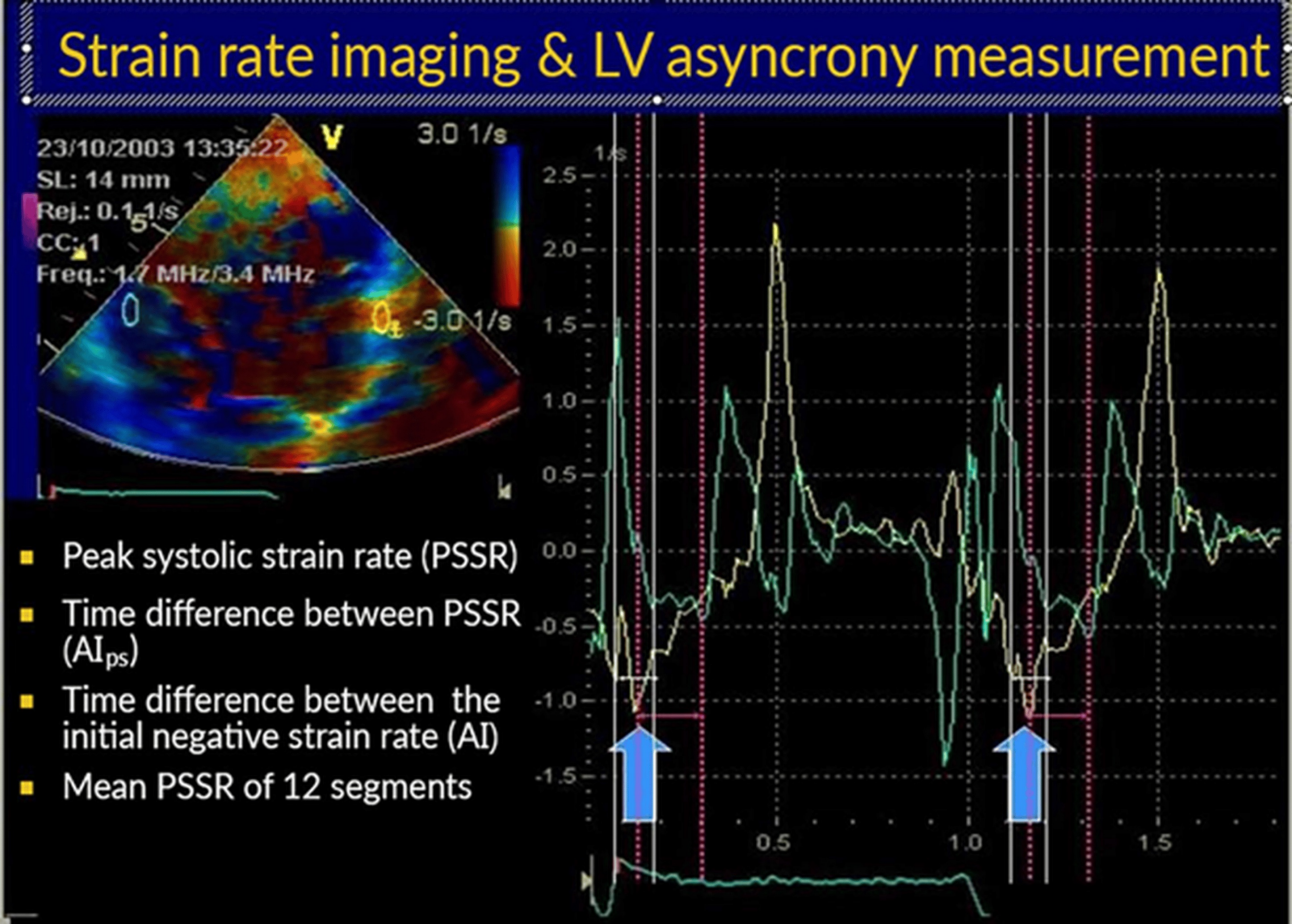
Measurement of asynchrony index LV: left ventricular

The asynchrony index is calculated by comparing the time required for the maximum systolic strain values of two separate myocardial segments or the strain values measured during isovolumic contraction and calculating the difference. In our study, the difference between the isovolumic active contraction timings determined according to the strain values of the lateral wall and the interventricular septum was taken as the asynchrony index (Figure 4).

Statistical analysis

The relationship between left ventricular dP/dt and ERO was evaluated by linear regression analysis. Data were expressed as mean ± SD and p value < 0.05 was considered significant. Calculations were made in a computer environment using SPSS version 11.0 (IBM Corp., Armonk, NY, USA). Data obtained with biventricular pacemaker on, and off times were analyzed by Wilcoxon test. Reproducibility of measured PISA and Doppler time intervals was evaluated by the mean differences between the values obtained from two independent measurements taken by two separate observers in the first 10 consecutive patients (inter-observer variability) and two independent measurements taken at different times in each patient by a single observer (intra-observer variability).

## Results

Baseline characteristics

Eighteen patients with severe left LV systolic dysfunction were included in our study. Their median age was 61 years and 12 of them were men. The patients’ mean LV ejection fraction was 22±5.6%. Thirteen patients were in NYHA functional class II and five patients in NYHA III. All patients had CRT with biventricular pacemakers. Additional information on baseline characteristics is listed in Table 1.

**Table 1:** Demographical and clinical data of all subjects. NYHA: New York Heart Association, LV: left ventricular, ACE: angiotensin-converting enzyme

Characteristic	n=18
Age, y	61±10
Male gender, n (%)	12 (66%)
NYHA II/III, n	13/5
İschemic cardiomyopathy, n	6
Nonischemic cardiomyopathy, n	12
QRS duration, ms	170±25
LV end diastolic volume, ml	288±87
LV end systolic volume, ml	227±80
Ejection fraction, %	22±5.6
ACE inhibitors	14 (77%)
Beta-blockers	15 (83%)
Spironolactone	12 (66%)

We measured LV dP/dt in 18 patients in their own rhythm and during biventricular stimulation. It decreased significantly in 10 patients when the biventricular pacemaker was interrupted (838±190 vs 444±72, p<0.01). To restore LV dP/dt to the levels observed during pacing, these patients received incremental dobutamine infusions at doses of 5, 10, 20, and 40 mcg/kg/min, with the infusion discontinued upon achieving the target dP/dt. However, in eight patients the LV dP/dt detected when the pacemaker was on (mean dP/dT=603±134) did not show a significant change when it was interrupted (dP/dt=592±156, p=0.679). The mean asynchrony index was 31±9 ms during the biventricular stimulation, however increased to 52±20ms after pacemaker deactivation showing a statistical significance (p < 0.0001).

Under comparable LV performance metrics (LV dP/dt, transmitral pressure difference), whether achieved through dobutamine infusion or maintained at pacing levels following pacemaker interruption, we observed a statistically significant reduction in ERO and RV during biventricular pacing across all patients (p<0.001 and p=0.003, respectively). The duration of mitral regurgitation varied, averaging 458±61 ms during pacing and increasing to 482±79 ms upon pacemaker deactivation, though this change demonstrated only weak statistical significance (p=0.046) (Table 2). Intraobserver variability for Doppler time intervals and velocities was calculated as 4±3% and 4±2%, respectively, while interobserver variability was 6±4% and 5±3%, respectively. For regurgitant volume calculations, intraobserver variability was 7±6%, and interobserver variability was 5±3% (Table 2).

**Table 2:** Detailed echocardiographic data of the entire study LV, left ventricle; PISA, proximal isovelocity surface area; FMR, functional mitral regurgitation; VTI, velocity-time integral; EROA, effective regurgitant orifice area; RV, regurgitant volume.

	Pacemaker on	Pacemaker abruptted (systolic performance parameters did not change or increased with dobutamine infusion)	
Asyncrony index, ms	31±9ms	52±20ms	P<0.0001
LV dP/dt	845±204mmHg/s	829±223mmHg/s	P=0.559
Maximal transmitral pressure gradient	111.4±25.6mmHg	102.7±19.5mmHg	P=0.077
PISA diameter	0.29±0.05cm	0.40±0.14cm	P=0.001
FMR VTI	172.8	169.7±35	P=0.678
FMR V max	5.20±0.62m/sn	5.12±0.60m/sn	P=0.645
FMR EROA	0.037±0.016cm^2^	0.076±0.049cm^2^	P<0.0001
FMR RV	6.64±2.07ml	14.39±6.69ml	P=0.003
FMR duration	458±61ms	482±79ms	P=0.046
Diastolic filling time	304±52ms	293±46ms	P=0.151

## Discussion

The findings of our study provide critical insights into the broader implications of CRT in managing FMR among DCM patients. Traditionally, CRT has been associated with global improvements in LV function, leading to a reduction in FMR. However, our study suggests that the benefits of CRT extend beyond global systolic performance enhancements. By targeting the timing of specific myocardial segments, particularly those associated with papillary muscle function, CRT can more effectively reduce FMR, thereby offering a refined therapeutic approach. Even though many studies have shown similar findings by strain imaging [13-15], to the best of our knowledge, this is a unique study evaluating the contribution of the local resynchronization to FMR degree under similar LV systolic indexes.

FMR is a common finding in patients with DCM as a complication of impaired LV systolic functions and remodeling. It is associated with progression of symptoms of heart failure, a higher rate of rehospitalization, and a poor prognosis [18]. The presence of moderate or severe FMR is related with a three-fold increased risk of heart failure and a 1.6-fold increased risk of death at five-year follow-up [17].

FMR is caused by the interaction of various mechanisms. The existence of local or global LV remodeling with changes in the geometric relationship between the ventricle and the MV apparatus which causes restricted leaflet motion known as "incomplete mitral leaflet closure," is a requirement for the development of MR [4]. The apical and posterior displacement of the papillary muscles because of remodeling causing systolic tenting with coaptation point away from the annulus, is the main determinant of FMR [2-4]. This restricts the movement of the leaflets and increases the degree of force required for mitral valve closure [2,4] which is the systolic transmitral pressure gradient between the LV and the left atrium. In LBBB, mechanical dyssynchrony not only diminishes the mitral valve's closing forces due to impaired left ventricular contraction but also predisposes to diastolic mitral regurgitation as a result of atrioventricular dyssynchrony. Impaired contraction of the papillary muscles itself and uncoordinated regional LV activation in the segments supporting the papillary muscles further increase tethering by provoking geometrical changes in mitral leaflets [9-13,19].

CRT increases LV dP/dt and hence the transmitral pressure difference and reduces FMR by counteracting the effect of tenting forces that impair mitral valve closure. In the study by Breithardt et al., an increase in the transmitral pressure difference was achieved both in peak systole and isovolumic contraction phase with CRT with an acute reduction of the midsystolic mitral valve tenting area and decrease in ERO of FMR from 25±19 mm2 to 13±8 mm2 [8]. According to a study by Soyama et al., the dyssynchrony determined by strain imaging between myocardial segments adjacent to papillary muscles is one of the causes of FMR [9]. Guler et al. [12] evaluated direct strain analysis of the papillary muscles themselves by assessing the difference in the time to peak systolic longitudinal strain between anterolateral and posteromedial muscles and they found that significant papillary muscle dyssynchrony (>30 ms) was the only independent predictor of moderate to severe FMR.

Ypenburg et al. [20] conducted a six-month follow-up evaluation of 25 patients who showed a reduction in FMR during the acute phase following CRT. At six months’ follow-up, the improvement in LV volumes and LV function was even more pronounced, with evidence of significant LV reverse remodeling. An acute deterioration of these parameters, except for LV end-diastolic volume, occurred during interruption of CRT at six months’ follow-up with the loss of papillary muscles resynchronization and recurrence of FMR. In our study, similar findings were observed in 10 patients when the biventricular pacemaker was interrupted, as significant decreases occurred in the dP/dt and transmitral pressure differences of the patients, while the duration of mitral regurgitation, EROs and RVs increased. Subsequently, with the dobutamine infusion after the pacemaker was turned off, the dP/dt value and transmitral pressure gradient similar to pacing level were obtained in 10 patients. However, in eight patients, there was no statistically significant change in LV dP/dt or transmitral pressure gradient when the pacemaker was interrupted (p=0.679). It is thought that this observation may be attributed due to the chronic reverse remodeling of the LV due to CRT. By enhancing reduction in LV size and sphericity, improving mitral valve function with the reduction in tethering forces, enhancing myocardial contractility and reducing myocardial stiffness, CRT not only improves global LV function but also specifically targets the structural and functional abnormalities that contribute to FMR. Therefore, the absence of significant changes in LV dP/dt or transmitral pressure gradient in some patients following pacemaker interruption could be attributed to the sustained benefits of chronic reverse remodeling. These patients may have achieved a more stable and efficient cardiac function that persists even in the absence of active CRT, thereby preventing the recurrence of severe FMR.

The remarkable finding of our study is that, despite similar LV dP/dts and transmitral pressure gradients, biventricular stimulation resulted in significantly lower EROs and RVs of FMR in all 18 of our patients. This data enables us to assume that the significant reduction in FMR during biventricular pacing state can be ascribed to an improved coordination of the LV wall underlying the papillary muscles with resynchronization in addition to improved systolic function.

Limitations

The main limitation of our study was the small sample size conducted at a single center. The primary objective of our study was to focus on the specific mechanistic contribution of local myocardial synchronization, particularly in segments supporting the papillary muscles. This selective approach allowed us to investigate the precise role of local segmental synchronization in mitigating FMR, an aspect that is often overlooked or underexplored in larger studies. While we acknowledge the limitations of a small sample size, this design provided a more detailed understanding of CRT’s localized effects on mitral valve function.

Furthermore, we recognize the need for larger-scale studies to validate these findings across broader populations. However, we believe our results contribute valuable insights into the mechanisms underlying CRT’s efficacy.

## Conclusions

This study demonstrates CRT significantly reduces FMR in DCM patients by primarily correcting dyssynchrony between papillary muscle-supporting myocardial segments, independent of global LV systolic improvement. These findings underscore the critical role of local myocardial synchronization in CRT's efficacy, highlighting the need for further research to validate these results and refine CRT strategies for enhanced FMR reduction.
